# Immediate or delayed retrieval of the displaced third molar: A review

**DOI:** 10.4317/jced.55379

**Published:** 2019-01-01

**Authors:** Dario Di Nardo, Giulia Mazzucchi, Marco Lollobrigida, Claudio Passariello, Renzo Guarnieri, Massimo Galli, Alberto De Biase, Luca Testarelli

**Affiliations:** 1 DDS, Ph.D. Department of Oral and Maxillo Facial Sciences, “Sapienza” University of Rome, Italy; 2 DDS. Department of Oral and Maxillo Facial Sciences, “Sapienza” University of Rome, Italy; 3 DDS. Department of Public Health and Infectious Diseases, “Sapienza” University of Rome, Italy

## Abstract

**Background:**

The displacement of a third molar is a rare occurrence, but it could lead to serious and/or life threatening complication. Aim of this review is to understand the most correlated causes of displacement and the possible solutions proposed in literature to avoid and solve this complication for maxillary and mandibular third molars at the appropriate time.

**Material and Methods:**

A search for “third molar displacement” was performed by using Pubmed database. Articles referred to soft tissues displacement, from 1957 to 2018, were included in the review. The references lists of all eligible articles were examined and additional studies were added to the review only if indexed on Pubmed. All the articles on maxillary sinus displacement and the dislocation of dental fragments or surgical equipment were excluded.

**Results:**

From a total of 134 results, 68 articles were examined for satisfying inclusion criteria. 18 articles were excluded because not inherent with the topic; 19 articles on infratemporal space, 11 on sublingual space, 9 on submandibular space, 11 on lateral pharyngeal space displacement were considered congruent for the review and included.

**Conclusions:**

The displacement of the third molar in deeper tissues could be avoided by the use of proper surgical procedures and instrumentarium. If displacement occurs, and the operator could not reach the tooth in safe conditions, the patient should be immediately referred to a maxillo-facial surgeon, because of the possibility of further displacement or the onset of hazardous or potentially fatal infections in vital regions.

** Key words:**Third molar, wisdom tooth, maxillary, mandibular, displacement.

## Introduction

The extraction of upper and lower third molars could lead to serious intra- and post-operational complications: the most common are nerve injuries, infections, hemorrhage, emphysema, prolapse of the buccal fat pad, mandibular fracture and alveolar osteitis. Swelling, pain, trismus and mild bleeding occur in approximately 10% of surgical removal of impacted third molars. Insufficient clinical and radiographic examination, lack of basic principles of surgery such as poor anatomic knowledge, inadequate flaps, decreased visibility and excessive or uncontrolled forces are often associated with the displacement of third molars into surrounding anatomic spaces (1-5).

The oldest articles on the displacement of the third molar available on Pubmed are dated 1957-1958 (6,7). The displacement most commonly involves the maxillary sinus and the submandibular space (8). Other sites of dislodgement are sublingual space, infratemporal space, pterygomandibular fossa, buccal space, lateral cervical space, pterygopalatine fossa, lateral pharyngeal space and superior ramus of the mandibula (9-11).

Objective of this review is to underline the most correlated causes of displacement and the possible solutions proposed in literature to avoid and solve this kind of complication for both maxillary and mandibular third molars at the appropriate time.

## Material and Methods

A search for “third molar displacement” was performed by using Pubmed database. Articles referred to soft tissues displacement, from 1957 to 2018, were included in the review. The references lists of all eligible articles were examined and additional studies were added to the review only if indexed on Pubmed. All the articles on maxillary sinus displacement and the dislocation of dental fragments or surgical equipment were excluded.

## Results

From a total of 134 results, 68 articles were examined for satisfying inclusion criteria. 18 articles were excluded because not inherent with the topic; 19 articles on infratemporal space, 11 on sublingual space, 9 on submandibular space, 11 on lateral pharyngeal space displacement were considered congruent for the review and included.

## Discussion

Displacement of the third molar in the infratemporal fossa ( Table 1).

**Table 1:**
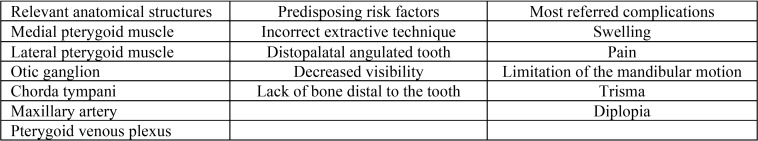
Infratemporal fossa’s main anatomical structures, displacement’s most correlated risk factors and associated complicatons.

The infratemporal region is located under the base of the skull between the pharynx and the mandibular ramus. It contains the medial and lateral pterygoid muscles, the otic ganglion, the chorda tympani, the maxillary artery and the pterygoid venous plexus. Molars are usually displaced through the periostium and located laterally to the lateral pterygoid plate and inferiorly to the lateral pterygoid muscle (4).

The displacement of maxillary third molars into the infratemporal fossa is usually associated with an incorrect extraction technique, distopalatal angulated tooth, decreased visibility during surgical removal or lack of bone distal to the tooth. Hence, an adequate surgical full-thickness flap, a congruent extractive force and the use of a distal retractor as the Laster retractor is highly recommended (12-15).

Clinically, a patient with a displaced tooth into the infratemporal fossa could be asymptomatic or present swelling, pain, limitation of the mandibular motion and trisma (2,3).

Conventional radiographic examination could disorient the operator due to the superimposition of anatomical structures: it could be necessary both panoramic, occipitomental, occlusal and lateral views. CT or CBCT examination should be encouraged due to their superiority in quality of the images and because they provide an exact localization of the displaced tooth (4,5,14,16,17).

Access to the infratemporal fossa is difficult and dangerous for the presence of vital structures running through it and the operator should not embark on potentially complicated and hazardous surgical procedures to retrieve the displaced tooth (5). An incautious attempt to remove the displaced tooth could lead to serious risk of hemorrage or neurologic injury and it may ultimately fail to retrieve the tooth, pushing it deeper into the tissues (18).

The first case of a third molar displaced in the infratemporal space was reported in 1977. In this case, an access was performed in the posterior wall of the maxillary sinus: even if no sinusitis occurred, a slight diplopia persisted after the removal (16).

In the 1986, Oberman *et al.* reported a case of displacement in the infratemporal fossa confirmed by a panoramic radiograph. In this case, the author attempted the retrieval of the tooth accessing the fossa through the maxillary sinus without success. The patient was followed up for 15 months during which no symptoms were referred and radiographs showed no visible lesions in the area of the displacement (17).

Extensive intraoral flaps were described for the retrieval of the displaced tooth due to the difficult access to the interested area and the lack of visibility: general anaesthesia could be often necessary (19). Other removal techniques described in literature were: intraoral flap with the resection of the coronoid process to extend the field of view (18), the access to the fossa through the posterior wall of the maxillary sinus (17) and the extraoral approach (20).

Patel and Down, extracted a wisdom tooth displaced in the infratemporal fossa under the zygomatic arch by using a fluoroscopic image intensifier for orthopaedic purpose and a standard Gillies approach to push the tooth through the oral incision. This approach was chosen after repeated and unsuccessful attempts under general anaesthesia and an on-table radiographic device with a marking needle (5).

Orr II described a case of immediate removal of a displaced tooth, lateral to the pterygoid plate and high in the infratemporal fossa in a young patient who was heavy sedated with diazepam, ketamine and meperidine. The tooth retrieval was perfomed by the aid of finger pressure and a 18-gauge spinal needle inserted from a superior direction, above the zygoma, posterior to the orbital rim (21).

The active navigation image guidance system proposed by Campbell *et al.*, allowed to real-time check the position of the displaced tooth, but surgical maneuvers were still conducted blindly (22).

The endoscopic approach is a less destructive procedure proposed for the retrieval of displaced teeth in the infratemporal fossa: it provides a constant direct view on the field and the displaced element. Battisti *et al.* removed a third molar displaced in correspondence of the pterygo-maxillary suture by the use of an endoscopic probe under general hypotensive anaesthesia through the primary surgical access in few minutes (23).

Delayed approaches (2-4 weeks) have been suggested in order to allow the formation of fibrous tissue which could stabilize the tooth, avoiding further displacement in the deeper areas. However, an antibiotic therapy should be always administered due to avoid infections in the area of the displacement (12,15,24,25).

Shahakbari R *et al.*, in the 2011, described for the first time the displacement of a mandibular third molar in the infratemporal space near the coronoid process. The displacement occured after the fracture of the lingual cortex during the extractive procedures. The patient complained limited mandibular movement and pain in the area of the attempted extraction. The removal was performed through a high ramus surgical access similar to a coronoidectomy incision and the patient showed an improved mandibular mobility immediately after the surgery (26).

Displacement in the sublingual space ( Table 2).

**Table 2:**
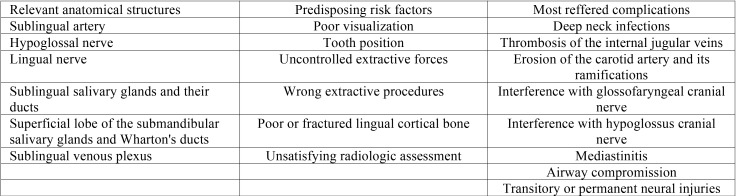
Sublingual space’s main anatomical structures, displacement’s most correlated risk factors and associated complications.

The sublingual area is a triangular virtual space, located in the floor of the mouth, above the mylohyoid muscle, under the free portion of the tongue. The sublingual space is limited by the muscle complex hyoglossus-styloglossus, and anteriorly, by the genioglossus muscle (27,28). The incidence of complications during the extraction of mandibular third molars is ~1,1%: the displacement of a tooth or its root fragment into the sublingual space is a very rare accident and it is related most with the age of the patient and the formation of the apex (29). The lower third molar can be displaced in the sublingual, submandibular and lateropharyngeal space in relation to the position of the mylohyoid ridge and the insertions of the mylohyoid muscle (30,31). The most referred symptoms are usually pain, swelling and trismus: in this case, immediate extraction should be performed. Bacterial contamination of the sublingual space may result in life-threatening complications: deep neck infections, thrombosis of the internal jugular vein, erosion of the carotid artery and its ramifications, interference with glossopharyngeal and hypoglossus cranial nerves, mediastinitis and airway compromission (27,29,32,33,34).

When an entire tooth is displaced in the submandibular space, it can migrate in deeper positions and it could be very difficult to reach and remove: risks related to surgery are due to the presence of important structures as sublingual artery, hypoglossal and lingual nerves, sublingual and submandibular salivary glands with their ducts (27,28,30). Transitory or permanent neural injuries were referred in literature and that kind of complications should be always considered before attempting surgery in this area (29,31).

Risk factors associated with lower third molars displacement into the sublingual space are related to tooth position, poor visualization of the surgical area, uncontrolled extractive forces, wrong extractive procedures, poor or fractured lingual cortical bone and unsatisfying radiologic assessment (29,34,35).

CT or cone beam CT are mandatory in order to exactly locate the fragment and they should be performed in concomitance with the surgery to avoid an excessive migration of the fragment in the period that follows the exam (29-31).

While some authors prefer to postpone surgery after 2-3 weeks to allow the formation of fibrous tissue that will stabilize the fragment, other authors prefer to immediate attempt the extraction to avoid infections, foreign bodies reaction or migration of the fragment in deeper areas (29,31,36).

An extended full thickness flap is recommended to allow a wide visualization of the surgical area and the use of proper instrumentation like periosteal elevators, flap retractors or finger pressure are mandatory to prevent further tooth’s displacement into adjacent areas. Sudden movements of the patient during the extractive procedures are involved in the further displacement of the fragment and they could be avoided under general anaesthesia (31,32). If the retrieval could be performed in relatively safe conditions, an intraoral surgical approach under regional anaesthesia (mandibular block) could be performed (27,34). Prophylactic broad-spectrum antibiotics should be always administered and referral to an oral or a maxillofacial surgeon is recommended in order to avoid the general dental practitioner being involved in potentially hazardous surgical procedures (1,27).

Displacement in the submandibular space ( Table 3.)

**Table 3:**
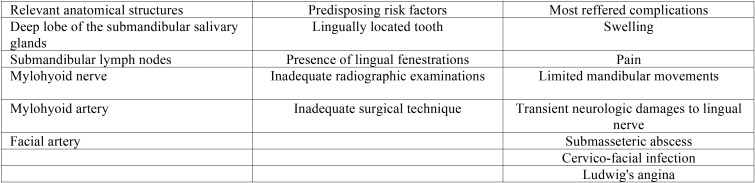
Submandibular space’s main anatomical structures, displacement’s most correlated risk factors and associated complications.

The submandibular space is located under the mylohyoid muscle and above the hyoid bone and the platysma muscle (37). The displacement of a tooth or a root’s fragment into the submandibular space is a very rare complication in the extraction of the lower third molar. This complication usually occurs when the tooth is located lingually, in presence of a fenestration of the lingual cortical plate with root exposure or when an inadequate radiographic examination or surgical technique is performed (exposure and/or ostectomy) (8,38-40). Furthermore, there are no posterior fascial borders limiting the sublingual and submandibular spaces and no fascial border separates these spaces from the inferior parapharyngeal space (37).

The submandibular displacement of a tooth may be accompanied by swelling, pain, limited mandibular movements, severe tissue damage, psychological distress, and medico-legal issues (8,41,42). It could be associated with permanent or transient neurologic damages to lingual nerve with paraesthesia of the lateral region of the tongue, numbness, intense pain, burning tongue and taste impairment (39,42). A case of submasseteric abscess occurred after 1 month from a submandibular displacement of an impacted lower third molar was also reported (43).

In case of unsuccessful attempt, if the retrieval is no more affordable, it is highly recommendable to immediate quit the intervention and quickly referral to a maxillofacial surgeon to avoid further displacement in deeper areas (37,38). The dental practitioner should inform the patient and all relevant informations should be provided due to prevent further delays and the progression to a more dangerous condition like a cervicofacial infection or Ludwig’s Angina (37-39).

The proper use of surgical instrumentation, extractive forces, with a exhaustive knowledge of the anatomical structures provided by a panoramic radiography and CT scans may prevent the iatrogenic displacement (37,41). Intraoral or extraoral finger pressure is considered useful in avoiding ligual cortex leakage and it could also aid the operator in finding and directing the displaced tooth to the way out (39,40,42).

Olusanya *et al.* reported a case of a 7 month delayed extraction of a left mandibular third molar displaced in the submandibular space. The patient referred a history of recurrent painful submandibular swelling. The swelling was firm, fibrotic and slightly tender, then the tooth was not palpable neither intraorally or extraorally. An extensive fibrosis nearby the submandibular salivary gland and the tooth stabilized it and prevented a further displacement in the cervical area. No panoramic radiography or CT scans were performed for the localization of the tooth: the only images obtained were a submentovertex and an oblique lateral view. Under general anaesthesia, a blunt dissection through an intraoral lingual mucoperiosteal flap and an extraoral submandibular incision was performed for the retrieval (38).

Solanki *et al.* used local anaesthesia for the removal of two lower third molars displaced in the submandibular region with an extraoral approach for the first case and a intraoral approach for the second one. In the second case, despite the extraoral approach was considered safer by the author, the patient explicitly refused any kind of cutaneous incision. However, in both cases systemic antibiotics were administered for 1 week and the postoperative course was uneventful (37).

Despite some authors consider the delay of the retrieval a favourable condition for the fixation of the fragment in a fibrous capsule, it may be assessed that submandibular displacement of a tooth or a fragment should be immediately encountered or addressed to a maxillofacial surgeon in order to avoid any further progression into deeper spaces and systemic antibiotics should be administered to prevent the onset of life-threating infections (41).

Displacement in the lateral cervical space ( Table 4).

**Table 4:**
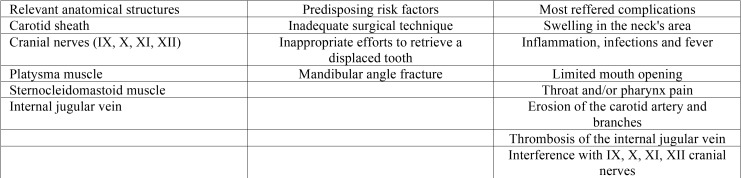
Lateral cervical space’s main anatomical structures, displacement’s most correlated risk factors and associated complications.

The lateral pharyngeal space extends from the base of the cranium on the sphenoid bone superiorly, to the hyoid bone inferiorly. It is divided into an anterior compartment that primarily contains the muscles, and a posterior compartment that contains the carotid sheath and numerous cranial nerves (44).

Inadequate surgical technique can cause displacement of the inferior third molar into the lateral pharyngeal space and may causes recurrent oral infections (45-48).

Occasionally, lower third molars displaced in the pterygomandibular space and upper third molars displaced in the infratemporal fossa, could be further displaced in the lateral cervical or pharyngeal areas (49-51). Further displacement from the pterygomandibular space into the lateral pharyngeal space could be provided by improper efforts to retrieve the tooth after its initial dislodgment. Kasatwar A, described an unusual case of displacement in the lateral pharyngeal space due to a mandibular angle fracture (52). Bobo, described a case of self-inflicted displacement of a maxillary third molar after that the patient attempted to extract the tooth by himself (49).

The most often reported symptoms are swelling in the area of the neck, inflammation, discomfort in swallowing and limited mouth opening; (44) Throat pain could occur immediately during the displacement then intermittent pharynx pain and fever could persist in delayed cases until the extraction being performed (45,50). A panoramic radiograph could help in the identification of the displacement, but it could not be sufficient even because the tooth is not always palpable, (44,45) only CT or Cone Beam CT are diriment for an exact localization of the displaced tooth (50).

Possible complications of delaying retrieval attempt are infections, foreign body reaction, thrombosis of the internal jugular vein, erosion of the carotid artery and its branches, and interference with cranial nerve IX through XII (52,53). To avoid possible life-threatening situations, a delayed retrieval approach is not recommended and an immediate intervention should be performed and systemic antibiotics should be always administered (54).Gay-Escoda *et al.*, described a lower right third molar in the lateral region of the neck, between the ptalysma and the sternocleidomastoid muscle, displaced after a 2 hours unsuccessful attempted extraction. The dentist did not informed the patient and the displacement was assessed only after 14 months of recurrent abscesses and inflammation involving the cervical area: those recurrent episodes are considered involved in the further displacement of the tooth. The extraction was performed extraorally by a cervical approach and the postoperative course was uneventful: the patient referred no symptoms even after 6 months from surgery (51).

Esen E *et al.*, described the retrieval via a transoral approach after tonsillectomy of a lower left third molar located at the anterior border of the lateral pharyngeal space, underlying the left tonsillar region. An approach via the tonsillar fossa was considered more convenient and less traumatic than an incision over the anterior border of the mandibular ramus with the tissutal dissection medial to the medial pterygoid muscle (53).

Lee described the extraction of an upper third molar displaced in the lateral pharingeal space, positioned medial to the medial pterygoid muscle and lateral to the superior constrictor muscle of the pharynx. The surgical approach was performed after months from the first attempt and a fibrous capsule was found around the tooth as reaction to the foreign body (50).

Ozalp, due to the patient’s refusal to extract a third mandibular molar displaced in deep neck tissues, obtained the improvement of the general conditions and the regression of inflammatory and infective conditions only by the administration of systemic antibiotics (endovenous 1 g amoxicillin and clavulanic acid for 14 days) (45).

## Conclusions

Even if there is no clear indication for the extraction of an asymptomatic molar, surgical removal may be reasonable in preventing serious complications and extraction should be performed at the appropriate time. Asymptomatic displaced or ectopic third molars which are not associated with any kind of lesion should be only followed-up and do not require treatment.

Otherwise, a displaced tooth is often correlated with morbidity and it should be removed due to prevent further displacement or potentially hazardous or life-threatening complications. If the general dentist is unable to reach the displaced tooth in safe conditions, he should quit procedures and immediately referral the patient to a maxillo-facial surgeon.
